# Cardiovascular Disease Risk in SMI Across Various Settings in a Semirural Area – a Study During COVID-19 Pandemic

**DOI:** 10.1192/bjo.2022.463

**Published:** 2022-06-20

**Authors:** Gaurav Mehta, Mahdi Memarpour, Aqsa Mohar, Mehtaab Mahal, Nehal Singh, Shweta Mehta

**Affiliations:** 1 University of Toronto, Toronto, Canada; 2 Southlake Regional Health Centre, Newmarket, Canada

## Abstract

**Aims:**

The risk for cardiovascular-related death is predicted to be higher in individuals with Serious Mental Illness (SMI) due to increased prevalence of common cardiac risk factors like smoking, physical inactivity, poor diet, substance use and hyperlipidemia among them.

**Methods:**

The aim of this retrospective study was to evaluate the physical health of patients with SMI in various settings- acute inpatient, tertiary care hospital and community.

We estimated the cardiovascular disease risk of schizophrenia patients with the aid of Framingham Risk Score (FRS) assessment tool, which can quantitatively predict both the heart age and 10-year CVD Risk percentage of patients aged ≥ 30 years. The clozapine to norclozapine ratio was compared with triglyceride levels, body weight, BMI, and fasting blood glucose in patients after treatment with clozapine. Southlake Regional Health Center's practice was compared with the national standards set by Diabetes Canada 2018 guidelines by conducting a clinical audit.

68 non-diabetic, patients aged ≥ 30 years with all the risk factor records for FRS assessment were selected from a cohort of 183 patients registered in the schizophrenia clinic of Southlake Regional Health Centre. The data were collected from patient records from the 75 patients registered with Assertive Community Treatment Team in Georgina, Ontario.

The sample size of the study on inpatients was 49 participants from the acute psychiatry ward consisting of 28 females and 21 males during the month of November 2021.

**Results:**

Males, on average, were found to have an intermediate 10-year CVD risk (~11.2%; FRS total points: 11.27) in comparison to females who, on average, had a low 10-year CVD risk (~7.3%; FRS total points: 11.19). 26% of the patients using FRS were calculated to be at high risk and 28% with intermediate risk of developing a CVD. The average heart age of the sample patients was 60 years, which was 9 years higher than the total average age (51 years). The investigated biomarkers of Hemoglobin A1C, triglycerides, and glucose serum concentration were examined graphically, separated into categories of the ratio measurements of 0–2, 2–3, and 3+. For all biomarkers, lower values were more desirable. Triglycerides were the lowest in the 3+ ratio category. Hemoglobin A1C and glucose serum concentration were lowest in the 0–2 ratio category.100% of patients with diabetes had their blood sugar levels measured and 66.67% were referred to an endocrinologist. In patients without diabetes, 91.30% had their blood sugar levels measured, 39.13% had their HbA1C levels measured, and 6.52% had neither their HbA1C, nor their blood sugar levels measured.

**Conclusion:**

Cardiovascular complication can be one of the leading causes of death in the next 10 years among schizophrenia patients due to age, poor lifestyle choices, and current estimations via the FRS assessment tool. Further studies need to be conducted with a larger sample size and more recent data to examine any adverse lifestyle changes in schizophrenia patients during the pandemic, which could have negatively influenced their cardiovascular health. It is recommended that doctors weigh the risks vs benefits of prescribing clozapine to patients with high triglyceride levels.

